# Exploring the effects of cerebellar tDCS on mental rotation

**DOI:** 10.1007/s10339-026-01355-3

**Published:** 2026-07-30

**Authors:** Angelica De Sandi, Fabiana Ruggiero, Denise Mellace, Angelica Marfoli, Carlo Manzoni, Natale Vincenzo Maiorana, Alice Tomaselli, Carmelo Campo, Maurizio Vergari, Benedetta Ticozzelli, Laura Mattiolli, Filippo Cogiamanian, Alberto Priori, Roberta Ferrucci

**Affiliations:** 1 Foundation IRCCS Ca’ Granda Ospedale Maggiore Policlinico, Milan, Italy; 2 Department of Oncology and Haemato-Oncology, University of Milan, Milan, Italy; 3 Aldo Ravelli Research Centre, Department of Health Science, University of Milan, Milan, Italy; 4 ASST-Santi Paolo e Carlo Hospital, Milan, Italy

## Abstract

Transcranial direct current stimulation (tDCS) on the prefrontal and posterior parietal cortex can enhance mental rotation (MR) performance, whereas little is known about cerebellar tDCS (ctDCS). This study aimed to evaluate effects of ctDCS on a MR task. Fifty-five healthy subjects were randomly assigned to active-ctDCS (1.5 mA; 20 min; *n* = 28) or sham-ctDCS (*n* = 27). Participants performed a MR task before (T0) and after (T1) stimulation. In each trial, two shapes appeared on a computer screen, one randomly rotated by 0, 45, 90, or 135 degrees. Subjects pressed the A or L key to indicate whether the shapes were identical or mirrored. A linear mixed model was run on the reaction times (RTs) recorded in each trial. Percent change in RTs (%RT) from T0 to T1 was also computed and analyzed using a second mixed-effects model with planned contrasts. A significant main effect of Time revealed RTs decreased from T0 (M = 3079.35, SE = 102.27) to T1 (M = 2565.36, SE = 102.27). No significant main effects or interactions involving Group were observed in either RT or %RT models. Planned contrasts revealed a single significant difference between groups in the 0° same condition, with the ctDCS group showing a greater reduction in %RT compared to the sham group. No other significant effects were found. ctDCS did not produce a global improvement in MR but showed selective effects under minimal spatial transformation demands. Further studies with different stimulation protocols are needed to clarify its role.

## Introduction

Mental rotation (MR) is the ability to mentally manipulate two-dimensional (2D) and three-dimensional (3D) objects (Shepard and Metzler 1971). Specifically, object rotation refers to the mental transformation of an external object in space (Shepard and Metzler 1971) relying on an allocentric strategy. An alternative strategy is the egocentric one, which consists in mentally transforming one’s own body representation, imagining it as moving relative to the environment (Parsons, 1987). These strategies rely on partially distinct cognitive and neural processes and may be differentially engaged depending on stimulus type and task demands (Ganesh et al. 2015; Perruchoud et al. 2016).

Much early MR research focused on humans’ ability to make a decision about the congruence of one rotated object with another. Shepard and Metzler (1971) first discovered that the time required to decide about the similarity of two rotated objects depends on the angular disparity between the two objects. This classic mental rotation task involves rotating an object relative to its internal axes. In contrast, mental rotation of body parts results in the transformation of an egocentric frame specified by the axes of the body (Parsons, 1987; Wraga et al. 2000).

Since it was first proposed by Shepard and Metzler (1971), numerous concepts have been developed to assess this aspect of visuospatial perception. One of the most common strategies for assessing MR is to determine the congruence (same/mirrored) of two stimuli that are rotated relative to each other, such as three-dimensional (3D) abstract shapes made of blocks (Shepard and Metzler 1971) or hands (Bode et al. 2007).

Several EEG, PET and fMRI studies investigated the neural correlates of MR, and showed that the MR network included activations in multiple cortical areas in both hemispheres (bilateral in the inferior and superior parietal lobes, bilateral in the left precentral gyrus, bilateral in the inferior Frontal gyrus, bilateral middle frontal gyrus, supplementary motor area SMA, left insula, inferior and middle occipital gyrus bilaterally) and cerebellum bilaterally (Hawes et al. 2019; Searle and Hamm 2017; Tomasino and Gremese 2016).

A systematic review (Veldema et al. 2021) aimed to investigate whether non-invasive brain stimulation effectively supports this relevant visuo-spatial ability and found that it is effective in modulating MR. In particular, studies have confirmed the effect of transcranial direct current stimulation (tDCS) in the prefrontal and parietal areas, showing improved performance on hand rotation tasks and recording faster reaction times (RTs) after stimulation (Tabrizi et al. 2019; Zhu et al. 2021).

The cerebellum has been increasingly recognized as contributing to spatial information processing even in the absence of overt motor activity, such as during mental rotation tasks. Anatomical, imaging, and clinical findings indicate that the cerebellum is involved in cognitive and affective functions as well as motor control (Balsters et al. 2013; Schmahmann and Sherman 1997; Stoodley et al. 2012). Neuroimaging findings suggested a functional topographical organization in the cerebellum with partially segregated regions supporting motor, cognitive, and affective functions (Stoodley 2012 a; Stoodley and Schmahmann 2016). More recent task-based functional mapping studies have further refined this view, showing that distinct cerebellar subregions are differentially engaged across multiple cognitive domains (King et al. 2019).

A fMRI study conducted by Stoodley et al. (2012) where participants were asked to judge whether a single rotated letter was displayed in its normal or mirror-image position found cerebellar activation during the task.

However, there are still few studies that have investigated the effects of non-invasive stimulation of the cerebellum in MR. Therefore, we explored how cerebellar tDCS affects MR performance, particularly in terms of reaction times (RTs).

We predicted that reaction times would improve from before tDCS (T0) to after tDCS (T1) and hypothesised that this improvement would be more pronounced in the group with cerebellar tDCS than in the sham stimulation group, supporting the proposed involvement of the cerebellum in MR.

Furthermore, based on previous evidence suggesting that performance in MR varies as a function of stimulus characteristics, particularly stimulus type and angular disparity (Tomasino and Gremese 2016; Shepard and Metzler 1971), and considering that neuromodulatory effects are known to be state- and task-dependent (Miniussi et al. 2013; Krause and Cohen Kadosh 2014), we hypothesised that stimulation effects might differ across stimulus types and rotation conditions.

## Materials and methods

### Subjects

Fifty-five healthy subjects (32 women and 23 men, aged 20–38; Table 1) volunteered to participate in this study. Participants had normal or corrected-to-normal vision and no history of neurological or psychiatric disease, nor were they taking medications that affect the central nervous system. The number of participants was calculated through a power analysis performed with GPower software, using a large effect size (f = 0.40) for a 2 × 2 design (group × time) with 80% power, considering an alpha level of 0.05.

Written informed consent was obtained from all participants before inclusion in the study. The study was approved by the Institutional Review Board and conducted in accordance with the Declaration of Helsinki.

### Experimental protocol

Participants were randomly assigned to one of two stimulation conditions (Table 1): active cerebellar stimulation (a-ctDCS, *n* = 28); sham cerebellar stimulation (s-ctDCS, *n* = 27). Participants were blind to the type of tDCS applied.

**Table 1:** Demographic features of the fifty-five participants treated with cerebellar and sham tDCS. Values are presented as means ± standard deviation

Subject (*n*)	a-ctDCS group	s-ctDCS group
	28	27
Gender	13 M; 15 F	10 M; 17 F
Age	25.82 ± 3.36	27.63 ± 6.22
Education	17.21 ± 1.1	17.11 ± 2.06

*Notes.* a-ctDCS: cerebellar transcranial direct current stimulation group; s-ctDCS: sham transcranial direct current stimulation group; F: female; M: male

**Fig. 1:**
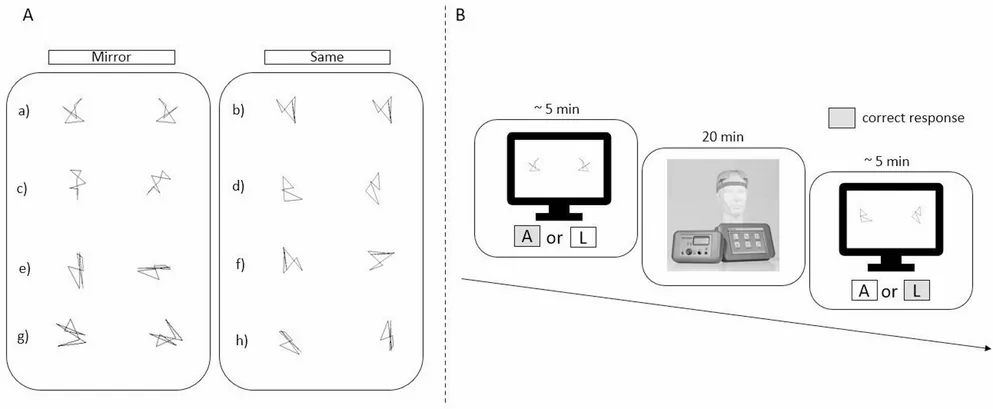
Experimental procedure. **A** Examples of mirror or identical images shown on a computer screen: mirror images rotated by 0 degrees (a); identical images rotated by 0 degrees (b); mirror images rotated by 45 degrees (c); identical images rotated by 45 degrees (d); mirror images rotated by 90 degrees (e); identical images rotated by 90 degrees (f); mirror images rotated by 135 degrees (g); identical images rotated by 135 degrees (h). **B** An example of one trial showing the sequence of events and timing. “A” and “L” refers to correct response buttons

A mental rotation task was administered using a computer-controlled procedure (Wadsworth CogLab software Publishing, Belmont, CA, USA) before (T0) and after (T1) tDCS to assess MR ability in all subjects (Fig. 1).

### tDCS

tDCS was administered with an electrical constant direct current stimulator (HDCstim, Newronika, Italy) using a pair of rubber electrodes in a 5 × 7 cm^2^ saline-soaked surface sponge. Active stimulation involved delivering a 1.5 mA direct current bilaterally over the cerebellum for 20 min per session, with a 10-second ramp-up and ramp-down period. The subjects in the sham group underwent the same procedure, but the current was applied only for the first 10 s. They experienced the initial itching sensation at the beginning of the stimulation, but no current was delivered afterward.

Participants were randomly assigned to one of two kinds of stimulation, which were designed before starting the experiment. In the a-ctDCS group the active electrode was positioned on the midline 2 cm below the inion with its lateral edges about 1 cm medial to the mastoid apophysis (above the cerebellum) and the reference electrode above the right arm. This montage has already been employed in past studies of cerebellar tDCS (Ferrucci et al. 2012, 2013). In the sham group, electrodes were placed in the same position as in the active condition, delivering non-effective stimulation that served as a control.

### Mental rotation task

Participants were tested in a quiet room sitting comfortably in a chair at approximately 80 cm from a computer monitor. The mental rotation task (Wadsworth CogLab software Publishing, Belmont, CA, USA) [33] was performed before (T0) and after (T1) tDCS stimulation. In each trial, two abstract shapes were displayed simultaneously next to each other on the left and right sides of a computer screen. Subjects were asked to press the A or L key on a computer keyboard to indicate whether the shapes, one of which was rotated relative to the other by 0, 45, 90, or 135 degrees, were mirror images or identical. The subjects were encouraged to mentally rotate the second stimulus to align it with the first (e.g., “as a consequence of an external force rotating the object”) (Barnes et al. 2000; Tomasino and Gremese 2016). The task included 80 stimulus pairs, which were presented in randomized order with respect to both rotation angle and stimulus type. Each session (T0 and T1) lasted approximately 5 min. The RTs of each participant were recorded during the task to assess performance.

### Statistics

RTs were first checked for linear model assumptions by assessing skewness and kurtosis values (judged as abnormal if > |1| and |3|, respectively) and by visually inspecting their histogram and Q-Q plot. As being judged to distribute normally, a linear mixed model was run on RTs which addressed, as predictors, Group (i.e., sham vs. cerebellum), Time (i.e., pre- and post-stimulation – T0 and T1, respectively), stimulus Type (i.e., same vs. mirror) and Condition (i.e., 0, 45, 90 and 135 degrees), as well as each possible interaction terms between them. Only main and interaction terms involving Group and Time were taken into account for this study, as the only ones of interest. Subjects were addressed as the cluster, with a random intercept being fitted. No random slopes were estimated for either main or interaction terms. Significant main terms were decomposed via Bonferroni-corrected post-hoc comparisons. To further investigate changes in performance, percent change scores in reaction times (%RTs) were computed as [(T1 − T0) / T0] × 100. An additional linear mixed-effects model was then fitted with %RT as the dependent variable Group (i.e., sham vs. cerebellum), stimulus Type (i.e., same vs. mirror), and Condition (i.e., 0, 45, 90 and 135 degrees), along with all their interactions, were included as fixed effects, while subjects were modeled as a random intercept. Model residuals and dependent measure distribution were visually inspected via histogram and Q-Q plot to verify approximate normality. Planned contrasts were conducted on the estimated marginal means derived from the model, and p-values were adjusted using the Holm–Bonferroni correction to control for multiple comparisons across condition combinations. Analyses were run via jamovi 2.3 (the jamovi project, 2023). The significance threshold was set at α = 0.05.

## Results

The descriptive statistics for participants are presented in Table 2.

**Table 2:** Data for reaction times (RTs) in the mental rotation task are reported in milliseconds (ms)

Condition	Pre-Stimulation		Post-Stimulation	
	Same	Mirror	Same	Mirror
a-ctDCS 0° 45° 90° 135°	1991.07 ± 495.02 2631.50 ± 660.98 2890.92 ± 933.37 3121.30 ± 1073.63	2275.49 ± 651.89 2771.21 ± 706.94 2992.06 ± 960.80 2884.74 ± 825.81	1392.19 ± 234.15 2130.58 ± 760.98 2409.3 ± 906.67 2487.12 ± 896.64	1728.12 ± 481.61 2152.6 ± 670.54 2372.69 ± 833.86 2512.99 ± 918.28
s-ctDCS 0° 45° 90° 135°	2136.87 ± 1091.52 2993.42 ± 1240.45 3057.70 ± 1146.15 3330.82 ± 1248.65	2411.70 ± 993.86 3129.28 ± 1243.18 3354.46 ± 1480.99 3251.38 ± 1239.48	1721.96 ± 672.33 2331.69 ± 772.11 2571.37 ± 812.82 2823.07 ± 932.35	1932.20 ± 560.63 2541.93 ± 874.14 2624.32 ± 993.41 2614.93 ± 790.43

RTs were recorded in each trial before and after tDCS stimulation. All values are presented as means ± standard deviation

*Notes.* a-ctDCS: cerebellar transcranial direct current stimulation group; s-ctDCS: sham transcranial direct current stimulation group

Table 3 reports the full results of the linear mixed model. Significant main effect was detected for Time. As for Time, a significant, overall reduction was found from T0 (M = 3079.35, SE = 102.27) to T1 (M = 2565.36, SE = 102.27). No other either main or interaction terms of interest yielded significance.

**Table 3:** Results of the linear mixed model on reaction times

Term	F	df_num_	df_den_	*p*
Group	1.5880	1	53.0	0.213
Time	234.2478	1	795.0	< 0.001
Type	6.9516	1	795.0	0.009
Condition	133.2751	3	795.0	< 0.001
Group ✻ Time	0.0514	1	795.0	0.821
Group ✻ Type	0.1969	1	795.0	0.657
Time ✻ Type	0.2751	1	795.0	0.600
Group ✻ Condition	0.5232	3	795.0	0.666
Time ✻ Condition	0.2720	3	795.0	0.846
Type ✻ Condition	5.2127	3	795.0	0.001
Group ✻ Time ✻ Type	0.5274	1	795.0	0.468
Group ✻ Time ✻ Condition	0.4294	3	795.0	0.732
Group ✻ Type ✻ Condition	0.4874	3	795.0	0.691
Time ✻ Type ✻ Condition	0.5643	3	795.0	0.639
Group ✻ Time ✻ Type ✻ Condition	0.6748	3	795.0	0.568

To further investigate differential changes in performance, a linear mixed-effects model was conducted on %RTs. Consistent with the analysis on raw RTs, no significant main effects or interactions involving Group were observed (all *p* > .05; Table 4).

Planned contrasts on the estimated marginal means, adjusted using the Holm–Bonferroni correction, revealed a single significant difference between groups in the 0° same condition. Specifically, the cerebellar stimulation group showed a greater reduction in %RTs (EMM = − 0.27 ± 0.05) compared to the sham group (EMM = − 0.13 ± 0.05), *p* = .031 (Holm–Bonferroni corrected). No other contrasts were significant after correction.

**Table 4:** Results of the linear mixed-effects model on (%RT)

Term	F	df_num_	df_den_	*p*
Group	0.90	1	155.34	0.345
Type	2.08	1	371.00	0.150
Condition	1.56	3	371.00	0.199
Group ✻ Type	1.50	1	371.00	0.222
Group ✻ Condition	1.00	3	371.00	0.395
Type ✻ Condition	1.99	3	371.00	0.116
Group ✻ Type ✻ Condition	1.68	3	371.00	0.170

## Discussion

The present study investigated the effects of cerebellar tDCS on MR performance, assessing changes in RTs before and after stimulation. While previous research has demonstrated that tDCS on the prefrontal and posterior parietal cortex improves MR performance (Tabrizi et al. 2019; Zhu et al. 2021), the role of cerebellar tDCS in this cognitive task remains poorly explored.

In contrast to our hypothesis, results showed a general improvement in all groups after tDCS, with no significant differences between the active and control groups.

To account for these findings, the involvement of the cerebellum in MR may be task-dependent, with different cerebellar regions engaged based on task complexity and spatial processing demands. Accordingly, ctDCS could selectively influence certain aspects of cognitive processing, rather than universally enhancing all dimensions of MR. More specifically, the effects of ctDCS could be influenced by the type of task stimulus. The type of task and the modality of stimulus presentation influence the regional activation pattern (Weiss et al. 2009), therefore the results may vary depending on the type of task proposed (body parts, alphanumeric stimuli, abstract shapes). Neuroimaging studies (Kosslyn et al. 1998; Parsons et al. 1995; Porro et al. 1996) have found that MR of the hands, and presumably other body parts, activate brain areas involved in visuomotor control and in hand movement, including the cerebellum (Ganis et al. 2000). These findings suggest that the cerebellum may not be specifically specialized for processing rotated abstract figures, but rather for other aspects of visuospatial cognition.

Within this framework, the distinction between allocentric and egocentric MR strategies may provide a useful interpretation. The cerebellum has been consistently implicated in visuomotor control and in the internal simulation of body-related transformations, processes that are more closely aligned with egocentric representations (Kosslyn et al. 1998; Parsons, 1987; Stoodley and Schmahmann 2016). Accordingly, cerebellar contributions to MR may be more pronounced when tasks involve embodied transformations, such as the mental rotation of body parts, which require the simulation of one’s own movements in space (Ganis et al. 2000; Parsons et al. 1995). By contrast, MR of abstract objects predominantly relies on allocentric, object-based spatial representations and on fronto-parietal networks supporting spatial manipulation (Hawes et al. 2019; Kosslyn et al. 1998; Tomasino and Gremese 2016). Given that the present task involved abstract shapes rather than body-related stimuli, it may have engaged cerebellar mechanisms to a lesser extent.

Task-based functional mapping studies have shown that distinct cerebellar subregions, including lateral posterior areas such as Crus I as well as more anterior motor-related regions, are differentially involved in cognitive processing (King et al. 2019). Some of these regions, which have been implicated in visual and visuospatial tasks, may not have been optimally targeted by the stimulation montage used in the present study.

Moreover, recent evidence indicates that MR of abstract figures engages a distributed cortico-subcortical-cerebellar network rather than a single specialized cerebellar locus (Tan et al. 2025). From this perspective, the limited and condition-specific effects observed here may reflect the spatial specificity of cerebellar stimulation and the network-based nature of mental rotation, rather than the absence of cerebellar involvement in processing rotated abstract figures.

Despite the absence of significant main effects or interactions involving Group in both the RTs and %RTs models, planned contrasts revealed a single significant difference between groups in the 0° identical (same) condition, with the active stimulation group showing a greater reduction in RTs than the sham group. Importantly, this effect was restricted to a single condition and did not generalize across rotation angles. The 0° same condition involves minimal spatial transformation and relies primarily on visual discrimination and shape-based recognition processes rather than on the core computational demands of MR. This pattern suggests that cerebellar tDCS may selectively modulate perceptual or visuospatial comparison subprocesses involved in conditions with minimal spatial transformation demands, rather than broadly enhancing MR performance.

At the same time, the 0° same condition cannot be considered a pure shape recognition task, as participants were required to judge whether the two stimuli were identical or mirror-reversed, thereby engaging visuospatial comparison mechanisms. However, because the two shapes were already aligned in orientation and structurally identical, congruency could be detected through direct perceptual and structural matching, without necessarily requiring an active MR to achieve alignment.

Consistent with classic models of MR, response times increase with angular disparity between stimuli (Shepard and Metzler 1971), indicating that greater spatial misalignment entails stronger engagement of rotation processes. When angular disparity is absent, as in the 0° same condition, the demand for MR is minimized. Moreover, theoretical accounts propose that observers initially rely on perceptual identification and structural comparison, resorting to MR only when spatial misalignment prevents direct matching (Cooper and Shepard 1973).

Within this framework, the 0° same condition can be interpreted as predominantly relying on shape-based recognition and low-level visuospatial comparison processes, with only minimal requirements for actual MR. This perspective may explain why a differential effect between groups emerged specifically in this condition despite the absence of a generalized Group × Time interaction across the task. Such an interpretation is consistent with the proposed role of the cerebellum in optimizing perceptual and sensorimotor processing (Stoodley 2012 b) as well as with neuroimaging evidence indicating cerebellar involvement in visual pattern processing (Shulman et al. 1997; Tao et al. 2024). However, direct evidence specifically linking the cerebellum to shape recognition remains limited.

An alternative explanation is that the observed effect may reflect a spread of the electric field to occipital visual regions, including primary visual cortex (V1). Nonetheless, if V1 involvement were the primary mechanism, a more generalized effect across stimulus types and rotation conditions would be expected, rather than the condition-specific effect observed only at 0° rotation.

Our findings, although intriguing, should be viewed in the context of certain methodological aspects that could have influenced the results. One such factor is task repetition, as all participants performed the task across two time points (pre- and post-stimulation). The lack of a pre-training session may have contributed to a learning effect across all groups, making it difficult to isolate the specific impact of tDCS.

One key aspect is the stimulation protocol used in this study. Electrode placement, stimulation intensity, and duration may all affect the efficacy of cerebellar tDCS (Ferrucci et al. 2015). Consequently, various methodological variables for cerebellar tDCS need to be systematically evaluated. The effects of tDCS on the cerebellum are still not well understood, and there are no established reference standards for tDCS dosage and duration (Ferrucci et al. 2019).

A critical factor is the timing of post-stimulation assessment. We measured MR performance only immediately after tDCS and did not consider potential changes occurring after longer periods of time. Indeed, a previous study showed effects of tDCS modulation only after 35 min post stimulation but not immediately after (Ferrucci et al. 2008). It is possible that the control activity of the cerebellum is organized in a feed-forward architecture that implies intermediate learning steps: the cerebellum processes information during task execution, which is then used to control subsequent tasks (Ferrucci et al. 2008).

The location of cerebellar stimulation may also play a crucial role. Previous studies (Picazio et al. 2013a, b) suggest that the left cerebellar hemisphere plays a crucial role in MR, regardless of stimulus type (Picazio et al. 2013a, b). Since our study applied bilateral tDCS, a more localized stimulation over the left cerebellum could be a promising direction for future research.

While this study provides novel insights into the potential role of cerebellar tDCS in visuospatial cognition, certain limitations must be considered. First, the sample size may not have been large enough to detect robust effects on this specific task, potentially limiting the generalizability of the findings. Additionally, the lack of response accuracy measurements represents a methodological limitation. Cognitive strategies employed by participants can influence both speed and accuracy, therefore considering these measures is essential for optimal performance analysis (Glickman et al. 2005). Without accuracy data, baseline comparability between groups cannot be fully established. This limitation also precludes the possibility of determining whether the condition-specific effect observed in the 0° same condition was influenced by individual differences in baseline performance.

## Conclusion

Our results suggest that cerebellar tDCS does not globally improve MR performance but may exert a selective and condition-specific influence on low-level perceptual or visuospatial comparison processes. In particular, the effect observed in the 0° same condition, which involves minimal spatial transformation demands, points to a possible modulation of perceptual discrimination rather than of the core mechanisms underlying MR. While methodological factors, such as the absence of experimental pre-training, may have contributed to the observed improvements, the stimulation did not broadly impact spatial transformation processes, as evidenced by the lack of effects across rotated conditions.

Further studies with a larger sample, using control conditions and testing different stimulation protocols, will be essential to clarify the specific contributions of the cerebellum in visuospatial cognition and its potential role in specific perceptual subprocesses.

## Data Availability

No datasets were generated or analysed during the current study.
